# Disrupted brain functional network topology is associated with peripheral inflammation in unmedicated bipolar II depression

**DOI:** 10.1017/S0033291725100834

**Published:** 2026-04-01

**Authors:** Guixian Tang, Guanmao Chen, Pan Chen, Feng Chen, Jurong Wang, Zhenye Luo, Zhangzhang Qi, Shuming Zhong, Hengwen Yang, Hui Zhong, Yanbin Jia, Li Huang, Ying Wang

**Affiliations:** 1 Medical Imaging Center, First Affiliated Hospital of Jinan University, Guangzhou 510630, China; 2 Institute of Molecular and Functional Imaging, Jinan University, Guangzhou, 510630, China; 3 Department of Nuclear Medicine, The Tenth Affiliated Hospital, Southern Medical University (Dongguan People’s Hospital), Dongguan 523059, China; 4 Department of Psychiatry, First Affiliated Hospital of Jinan University, Guangzhou 510630, China; 5 Biomedical Translational Research Institute, Jinan University, Guangzhou 510630, China

**Keywords:** bipolar disorder, graph theory, inflammation, IL-8, rs-fMRI

## Abstract

**Background:**

Increasing evidences show that inflammation might be involved in bipolar disorder (BD), but the association between abnormal brain function and inflammation in BD is still unclear. In this study, we tried to explore the disrupted brain functional network topology, peripheral inflammatory cytokine levels, and their correlations in unmedicated bipolar II depression (BDII-D).

**Methods:**

In this study, 65 individuals with unmedicated BDII-D and 50 healthy controls (HCs) underwent resting-state magnetic resonance imaging scans. Graph theory analysis was performed to investigate the topological properties of the whole-brain functional connectome at both global and nodal levels. Besides, serum levels of 17 inflammatory cytokines were measured in both BDII-D and HCs. Correlations between topological properties, clinical variables, and peripheral inflammatory cytokine levels in BDII-D were calculated.

**Results:**

Compared with HCs, at the global level, BDII-D showed significantly higher 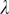 , decreased 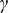 , 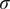 , *E_glo_*, and *E_loc_*; at the nodal level, BDII-D showed decreased *E_nodal_* in the right olfactory cortex, left pallidum, and vermis. Besides, BDII-D showed higher levels of interleukin-8 (IL-8), interleukin-10 (IL-10), and granulocyte colony-stimulating factor (G-CSF) compared with the HCs. In BDII-D, 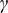 and 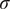 were significantly negatively correlated with the Hamilton Depression Rating Scale (HDRS) scores and number of episodes. Also, IL-8 level showed significant negative correlation with 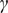 , 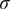 , and *E_nodal_* of the left pallidum in BDII-D.

**Conclusions:**

Reduced information segregation and integration, and lower nodal efficiency in the left pallidum were associated with proinflammatory cytokine IL-8 level and might contribute to severe depressive symptoms in unmedicated BDII-D.

## Introduction

Bipolar disorder (BD) is a chronic mental illness characterized by recurrent periods of mania and depression (BDI) or hypomania and depression (BDII) (Phillips & Kupfer, 2013). The depressive episodes in BDII are more persistent and lead to a higher burden of depression over time compared to BDI, because hypomania is less severe than mania but is easier to overlook (Baek et al., 2011). Genetic (Lee et al., 2011), metabolic (Nikolaus, Müller, & Hautzel, 2017), and neuroimaging (Abé et al., 2016; Caseras et al., 2013) investigations have demonstrated that BDI and BDII differ in their pathophysiological and neurobiological mechanisms. Moreover, inflammation in BD has been shown to be associated with cognitive dysfunction (Barbosa et al., 2018; Dickerson et al., 2013), abnormal mood states (mania and depression) (Fiedorowicz et al., 2015), illness activity (Kapczinski et al., 2011), and different clinical stages (Tatay-Manteiga et al., 2017). Given these findings, it is essential to further explore the role of inflammation in BDII.

Systemic inflammation and immune dysregulation have been considered to play a significant role in psychiatric disorders such as BD, major depressive disorder (MDD), schizophrenia, and obsessive-compulsive disorder (Najjar et al., 2013). Specifically, BD has been reported to be a disease associated with chronic inflammatory and immunological alterations mediated by cytokines’ cascades, cellular immune responses, oxidative factors, and hormone regulation (Altamura, Buoli, & Pozzoli, 2014). Cytokines could access the brain and influence many neurobiological processes known to be involved in depression, including neurotransmitter metabolism, neuroendocrine function, and neural plasticity (Miller, Maletic, & Raison, 2009), facilitating peripheral-to-central immune crosstalk (Walker et al., 2023). Cytokines can enter the central nervous system and trigger neuroinflammation through increased blood–brain barrier (BBB) permeability (Hu et al., 2024) and can also alter cerebrospinal fluid (CSF) composition by affecting the choroid plexus (Balusu et al., 2016), with previous studies identifying correlations between choroid plexus volume and peripheral cytokine levels in BDII-D (Cao et al., 2023). Previous review and meta-analyses have reported elevated levels of inflammatory cytokines such as interleukin-4 (IL-4), interleukin-6 (IL-6), interleukin-8 (IL-8), interleukin-10 (IL-10), and tumor necrosis factor-α (TNF-α) in BD (Goldsmith, Rapaport, & Miller, 2016; Modabbernia, Taslimi, Brietzke, & Ashrafi, 2013; Munkholm, Braüner, Kessing, & Vinberg, 2013; Solmi et al., 2021; Tsai, 2021), and cytokine profiles might differ across mood states and BD subtypes (Long et al., 2024; Munkholm et al., 2013). Moreover, cytokines have been robustly associated with psychiatric symptoms and regional brain abnormalities, including functional connectivity disruptions (*Chen et al., 2020*; Tang et al., 2021; Xiao et al., 2024), structural gray matter loss (Cao et al., 2024), and reduced white matter (WM) integrity (Cao et al., 2025) in BDII-D. In our previous study, the disruption of regional FC was inversely correlated with IL-8 level in BD II (Tang et al., 2021). However, the relationships between cytokine levels and whole-brain functional abnormalities remain unclear.

In recent years, there has been growing interest in understanding the complex relationships between inflammation and brain function, especially in the context of bipolar disorder. Traditional functional connectivity (FC) analysis has provided valuable insights into these relationships; however, it may not fully capture the intricate topological organization of the brain’s functional networks (Gong & He, 2015). Studying the topological organization can provide additional information on how different brain regions interact and coordinate their activities, which is crucial for understanding the pathophysiology of bipolar disorder. Graph theory-based analysis has been applied to resting-state functional magnetic resonance imaging (rs-fMRI) to study the features of complex brain networks (Bullmore & Sporns, 2009; Liao, Vasilakos, & He, 2017). In terms of graph theory, the human brain’s functional networks can be defined as a complex dynamic system consisting of functionally connected nodes (brain regions) and edges (functional connections between these brain regions) (van den Heuvel & Hulshoff Pol, 2010). This method derives various parameters that reflect the topological properties of the brain’s functional networks, such as clustering coefficient, characteristic path length, small-worldness, global efficiency, and local efficiency, at the global level, and nodal efficiency, at the nodal level (Bullmore & Sporns, 2012; Gozdas, Holland, & Altaye, 2019; Jiang et al., 2020; van den Heuvel & Hulshoff Pol, 2010; Zhou et al., 2017). For example, global efficiency reflects the overall efficiency of information transfer in the brain network, and nodal efficiency reflects the efficiency of information transfer in specific brain regions (Bassett et al., 2009). These parameters provide a more comprehensive understanding of the brain’s functional organization and its relationship with inflammation in BD. Previous studies using graph theoretical brain network analysis have reported topological alterations of functional networks in BD (Kim et al., 2013; Wang et al., 2017; Xia et al., 2019). For example, at the global level, BD II individuals showed increased characteristic path length and decreased global efficiency compared to the controls (Wang et al., 2017); at the nodal level, BD exhibited significant abnormalities and decreased nodal efficiency in the left ventral pallidum (Zhang et al., 2023). In MDD, significant correlations were observed between clinical symptoms as well as plasma IL-6 levels with whole-brain functional network connectivity (Liu et al., 2024), suggesting the potential role of whole-brain functional network disruption in the pathophysiological mechanisms of depression.

Existing studies have primarily focused on either the investigation of brain network alterations in BD (Kim et al., 2013; Wang et al., 2017; Xia et al., 2019), or on the exploration of peripheral inflammation in the context of BD using traditional methods without specifically linking it to the brain network topology (*Chen et al., 2020*; Tang et al., 2021; Xiao et al., 2024). To date, the link between peripheral inflammation and network-level dysfunction in BD remains unclear, particularly regarding whether alterations in inflammatory cytokines are correlated with critical brain network properties from topological analysis. In this study, we hypothesized that unmedicated BDII-D individuals would show abnormal global parameters such as greater characteristic path length, lower clustering coefficient, lower global efficiency, and decreased nodal efficiency compared to healthy controls (HCs). We also expected greater levels of inflammatory cytokines in unmedicated BDII-D. Therefore, we assumed that the abnormal global and nodal parameters would show correlation with peripheral cytokine levels (such as IL-8) and clinical variables in unmedicated BDII-D. By exploring these associations, this study aims to elucidate the potential interplay between peripheral inflammation and brain network disruptions in BDII-D, thereby contributing to a more comprehensive understanding of the pathophysiology of this disorder.

## Materials and methods

### Participants

Sixty-five unmedicated BDII-D individuals were recruited from the psychiatry department of the First Affiliated Hospital of Jinan University, Guangzhou, China. The BDII-D individuals were aged from 18 to 55 years old, to reduce the potential influence of age-related vascular lesions and late life multimorbidity (Ben Hassen et al., 2022). Participants were recruited between March 2018 and June 2022. The BDII-D participants were diagnosed based on the Diagnostic and Statistical Manual of Mental Disorders (Fifth Edition) (DSM-5). The enrolled participants were diagnosed by two experienced clinical psychiatrists (Y.J. and S.Z., with 27 and 10 years of experience in clinical psychiatry, respectively) based on the Structured Clinical Interview for DSM-IV Patient Edition (SCID-P). During a 3-day period before the neuroimaging acquisition, the 24-item Hamilton Depression Rating Scale (HDRS) and the Young Mania Rating Scale (YMRS) were used to evaluate the clinical state of BD individuals. Individuals who met the conditions of the 24-item HDRS total score more than 20 and YMRS less than 7 were recruited. Exclusion criteria were as follows: (i) currently suffering from any psychiatric disorder or associated symptoms; (ii) history of any organic or neurological brain disorder; (iii) history of any alcohol/substance abuse or dependence; (iv) history of use of any psychotropic medication, psychotherapy, or electroconvulsive therapy; (v) any physical illness demonstrated by clinical or laboratory examinations, or personal history; and (vi) pregnancy or postpartum depression, and any contraindication to magnetic resonance imaging (MRI) scanning. All the individuals have not received medication for at least 6 months at the time of the neuroimaging acquisition.

Fifty right-handed healthy volunteers were recruited in this study through local advertisements at the same period of time. All the HCs were chosen after a diagnostic interview (the SCID Nonpatient Edition) to eliminate the possibility of current or past history of psychiatric illness. Moreover, HCs with the following situations would be excluded from this study: history of psychiatric illness in first-degree relatives, or current or past significant medical or neurological illness.

This study was approved by the Ethics Committee of First Affiliated Hospital of Jinan University, China. All the participants signed informed consent forms after a full written and verbal explanation of the study.

### MR imaging data acquisitions

Neuroimaging data were acquired on GE Discovery MR750 3.0 T machine with the eight-channel phased-array head coil. All the subjects were scanned in supine, head-first positions, with cushions placed symmetrically on both sides of their heads to reduce movement. The subjects were told to relax and not close their eyes or fall asleep during the scan. At the end of the experiment, it was confirmed that none of the participants had fallen asleep. The rs-fMRI data were obtained by gradient-echo echo planar imaging sequence, and its parameters were as follows: time repetition (TR)/time echo (TE) = 2000/25 ms; field of view (FOV) = 240 × 240 mm^2^; flip angle = 90°; voxel size = 3.75 × 3.75 × 3 mm^3^; slice thickness/gap = 3.0/1.0 mm; matrix = 64 × 64; 35 axial slices covering the whole brain; and 210 volumes acquired in 7 min. Additionally, whole-brain, three-dimensional brain volume imaging (3D-BRAVO) sequence was used to collect brain structural data, and the parameters were as follows: TR/ TE = 8.2/ 3.2 ms; bandwidth = 31.25 Hz; flip angle = 12°; NEX = 1; slice thickness/gap = 1.0/0 mm; FOV = 240 × 240 mm^2^; matrix = 256 × 256; and acquisition time = 3 min 45 s. Also, conventional MRI data of all the participants were acquired to confirm the absence of any anatomic abnormalities of their brains, which were determined by two experienced neuroradiologists (G.C. and G.T.).

### Functional data preprocessing

The preprocessing was carried out using Statistical Parametric Mapping (SPM 12, http://www.fil.ion.ucl.ac.uk/spm/) and Data Processing & Analysis of Brain Imaging (DPABI_V3.0, http://restfmri.net/forum/DPABI) (Yan, Wang, Zuo, & Zang, 2016). For each subject, the first 10 volumes of the rs-fMRI dataset were removed; the remaining 200 volumes were slice timing corrected; and all images were realigned to the first image for head motion correction. Individuals who had more than 2 mm maximum displacement, 2°of angular motion, and 0.2 mm in mean frame-wise displacement were excluded (Jenkinson, Bannister, Brady, & Smith, 2002). Several spurious covariates (global signal, white matter, cerebrospinal fluid signals, and Friston-24 parameters) were removed from the data. The Diffeomorphic Anatomical Registration Through Exponentiated Lie algebra (Shen & Sterr, 2013) segment toolbox was utilized to create templates for spatial normalization to the Montreal Neurological Institute (MNI) space with 3 × 3 × 3 mm^3^ voxels, which was followed by signal linear trend removing and temporal band-pass filtering (0.01–0.1 Hz).

### Constructing brain functional networks

The brain functional network of each subject was constructed using the GRETNA toolbox (http://www.nitrc.org/projects/gretna/) (Wang et al., 2015) according to the automated anatomical labeling (AAL) template (Tzourio-Mazoyer et al., 2002). First, the AAL template was parcellated into 116 brain regions as the regions of interest (ROIs); each ROI was defined as a node of the network. The time series for each ROI was calculated by extracting and averaging the time series of all the voxels within the ROI. Second, to determine the edges of brain networks, for each subject, Pearson’s correlation coefficients were computed between the given ROI and the other 115 ROIs, and we repeated this process for all given ROIs. Then, a 116 by 116 symmetric partial correlation matrix (R) for each subject was obtained. Of note, in the matrix, because of detrimental effects on test–retest reliability and ambiguous interpretation (Fox, Zhang, Snyder, & Raichle, 2009; Murphy et al., 2009; Weissenbacher et al., 2009), negative correlations were excluded. Finally, binary graphs were obtained from the sparsity threshold from 0.1 to 0.4 with intervals of 0.01, which was used in subsequent analyses. Sparsity value was equal to the ratio of the actual total number of edges to the maximum possible number of edges.

### Small-world network properties

At each sparsity threshold, we calculated global and nodal network properties. Several global network properties involving the small-world parameters and network efficiency were computed. The small-world parameters included normalized clustering coefficient 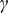 , normalized characteristic path length 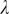 , and small-worldness 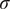 (the ratio of 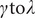 ). The network efficiency indexes included global efficiency *E*_glob_ and local efficiency *E*_loc_. We also calculated nodal efficiency *E*_nodal_, one of the parameters of nodal properties. Detailed interpretations of these properties are shown in the previous study (Rubinov & Sporns, 2010).

Thus, we applied five global parameters ( 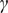 , 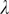 , 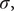 *E*_glo_, and *E*_loc_) and one regional nodal parameter (*E*_nodal_) to characterize functional network properties. For each network index, we further calculate the area under the curve (AUC) to obtain the summary scalar of the brain function network topology representation (Zhang et al., 2011).

### Inflammatory cytokine measures

Peripheral blood samples from 34 unmedicated BDII-D individuals and 30 HCs were collected in fasting states in the morning (all the participants without alcoholic beverages intake for at least 1 day before testing) and were processed (then frozen) by technicians. Details on processing blood samples can be found in the Supplementary Materials. Serum levels of 17 inflammatory cytokines, including interleukin 1β (IL-1β), interleukin 2 (IL-2), IL-4, interleukin 5 (IL-5), IL-6, interleukin 7 (IL-7), IL-8, IL-10, interleukin 12 (IL-12), interleukin 13 (IL-13), interleukin 17 (IL-17), granulocyte colony-stimulating factor (G-CSF), granulocyte-macrophage colony-stimulating factor (GM-CSF), interferon-γ (IFN-γ), monocyte chemoattractant protein-1/monocyte chemotactic and activating factor (MCP-1/MCAF), macrophage inflammatory protein 1β (MIP-1β), and TNF-α were measured using the Bio-Plex Human Cytokine 17-Plex panel in combination with the Bio-Plex Suspension Array System (Bio-Rad Laboratories Inc., Hercules, CA, USA) (Gauglitz et al., 2008). Bio-Plex Manager Software, version 6.1, was used for data acquisition (Tang et al., 2021).

### Statistical analysis

#### Comparison of demographic variables

Independent sample *t*-tests and chi-square tests were used to compare the demographic data between the BDII-D and HC groups using SPSS 19.0 software (SPSS, Chicago, IL, USA). All tests were two-tailed, and *p* < 0.05 was considered statistically significant.

#### Comparison of network parameters and cytokine levels

The Kolmogorov–Smirnov tests were used to determine whether network parameters including global network topological parameters ( 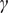 , 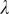 , 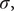 *E*_glo,_ and *E*_loc_), nodal parameter (*E*_nodal_), and cytokine levels including IL-1β, IL-2, IL-4, IL-5, IL-6, IL-7, IL-8, IL-10, IL-12, IL-13, IL-17, G-CSF, GM-CSF, IFN-γ, MCP-1, MIP-1β, and TNF-α fit within normal distributions. If the network parameters and cytokine levels exhibited normal distributions, the data would be expressed as the mean and standard deviation, and independent sample *t*-tests were used to compare data between two groups. If a normal distribution was not observed, the median values and interquartile range would be reported, and subsequent comparisons of network parameters and cytokine levels between groups were performed using the Mann–Whitney *U*-tests. To stabilize variance and normalize the distribution, all cytokine levels were normalized (base 10 log-transformed) before analysis. False discovery rate (FDR) was applied for multiple comparison corrections, and *p* < 0.05 after FDR correction was considered statistically significant.

#### Correlations between the network properties, inflammatory cytokines, and clinical variables

Partial Pearson correlation analyses (controlled for age, gender, and education) were performed to explore the relationships between network properties and log-transformed inflammatory cytokine levels that showed significant group differences after FDR corrections and their relationships with clinical variables in BDII-D. The clinical variables included the 24-item HDRS score, the YMRS score, onset age of illness, number of episodes, and durations of illness.

### Validation analyses

The complementary utility of binary and weighted functional network (Cole, Pathak, & Schneider, 2010) has been reported previously, as binary networks show strengths of reduction of the computational complexity and clearness of network metric definitions, while connectivity strength is taken into account in weighted networks (Wang et al., 2015). To determine whether the observed group differences in the topological parameters reflect true differences rather than artifacts, we repeated the network analyses using weighted graphs from the sparsity threshold from 0.1 to 0.4 with intervals of 0.01 to validate the main results obtained from binary graphs. Furthermore, we repeated the correlation analyses between weighted functional network topological parameters, clinical variables, and inflammatory cytokine levels to validate the correlation results of the main findings.

## Results

### Demographic and clinical characteristics

Demographic and clinical characteristics of all the participants are displayed in Table 1. No significant differences were found in age, sex, and education levels between the BDII-D and HC groups.

**Table 1.** Demographic data and clinical variables of the two groups

	BDII-D	HCs	*t/x* ^*2*^	*p* values
Number of subjects	65	50		
Age (years)	29.37 (9.46)	31.65 (10.86)	−1.207	0.230^a^
Gender (male/female)	28/37	24/26	0.277	0.599^b^
Education (years)	14.22 (3.96)	15.74 (4.44)	−1.940	0.055^a^
Age at onset (years)	25.62 (8.27)	n/a		
Number of episodes	2.96 (1.62)	n/a		
24-item HDRS score	27.65 (8.37)	n/a		
YMRS score	2.73 (2.72)	n/a		
Duration of illness (months)	41.98 (53.70)	n/a		

*Note:* Means (with standard deviations in parentheses) are reported unless otherwise noted.

Abbreviations: BDII-D, bipolar II depression; HCs, healthy controls; HDRS, Hamilton Depression Rating Scale; YMRS, Young Mania Rating Scale.

a The *p* values were obtained by independent sample t-tests.

b The *p* value for gender distribution was obtained by the chi-square test.

### Global and nodal parameters of the brain functional network

**Figure 1.**
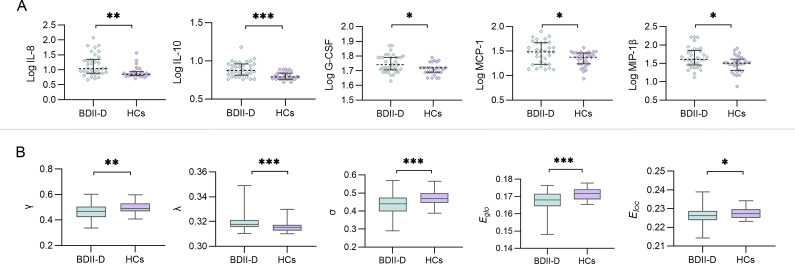
Group differences of the inflammatory cytokine levels and the global topological parameters between the BDII-D and HCs group. (A) The BDII-D group showed significantly higher levels of IL-8, IL-10, G-CSF, MCP-1 and MIP-1β compared to HCs (FDR corrected, *p* < 0.05). (B) The BDII-D group showed significantly higher *λ*, and significantly decreased *γ*, *σ*, *E_glo_*, and *E_loc_* compared to HCs (FDR corrected, *p* < 0.05). BDII-D, bipolar II depression; HCs, healthy controls; FDR, false discovery rate; IL-8, interleukin 8; IL-10, interleukin 10; G-CSF, granulocyte colony-stimulating factor; MCP-1, monocyte chemoattractant protein-1; MIP-1β, macrophage inflammatory protein 1β; *γ*, normalized clustering coefficient; *λ*, normalized characteristic path length; *σ*, small-worldness; *E_glo_*, global efficiency; *E_loc_*, local efficiency. Log, base 10 log-transformed. *: *p* < 0.05. **: *p* < 0.01. ***: *p* < 0.001.

Figure 1 and Table 2 show the results of global parameters (normalized clustering coefficient 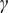 , normalized characteristic path length 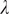 , small-worldness 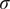 , global efficiency *E*_glob,_ and local efficiency *E*_loc_) for the BDII-D and HC groups. Statistical analyses revealed that the BDII-D group showed significantly higher 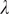 (*z* = −4.028, *p* < 0.001, FDR-*p* < 0.001) and significantly decreased 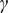 (*t* = −3.285, *p* = 0.001, FDR-*p* = 0.001), 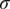 (*t* = −3.721, *p* < 0.001, FDR-*p* < 0.001), *E_glo_* (*z* = −3.769, *p* < 0.001, FDR-*p* < 0.001), and *E_loc_* (*t* = −1.993, *p* = 0.049, FDR-*p* = 0.049) compared to HCs.

**Table 2.** Statistical results for the global and nodal parameters between the BDII-D group and the HC group

Parameters	BDII-D	HCs	*t/z*	*p* values	FDR-*p*
**Global parameters**					
*γ*	0.4669 (0.0542)	0.4961 (0.0412)	−3.285	0.001^a^	0.001^a^
*λ*	0.3175 [0.3156–0.3210]	0.3154 [0.3126–0.3172]	−4.028	<0.001^b^	<0.001^b^
*σ*	0.4364 (0.0543)	0.4688 (0.0391)	−3.721	<0.001^a^	<0.001^a^
*E_glo_*	0.1680 [0.1643–0.1716]	0.1716 [0.1683–0.1741]	−3.769	<0.001^b^	<0.001^b^
*El_oc_*	0.2262 (0.0048)	0.2276 (0.0028)	−1.993	0.049^a^	0.049^a^
**Nodal efficiency**					
R Olfactory Cortex	0.1371 [0.1056–0.1552]	0.1557 [0.1460–0.1702]	−3.921	<0.001^b^	<0.001^b^
L Pallidum	0.1665 (0.0248)	0.1803 (0.0160)	−3.628	<0.001^a^	<0.001^a^
Vermis	0.0964 [0.0639–0.1326]	0.1294 [0.1014–0.1462]	−3.523	<0.001^b^	<0.001^b^

*Note:* Means (with standard deviations in parentheses) and median [25% quartile-75% quartile] are reported unless otherwise noted. BDII-D, bipolar II depression; HCs, healthy controls; FDR, false discovery rate; 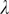 , normalized characteristic path length; 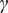 , normalized clustering coefficient; *σ*, small-worldness; *E_glo_*, global efficiency; *E_loc_*, local efficiency; R, right; L, left.

a The *p* values were obtained by independent sample t-tests.

b The *p* values were obtained by Mann–Whitney U tests.

**Figure 2.**
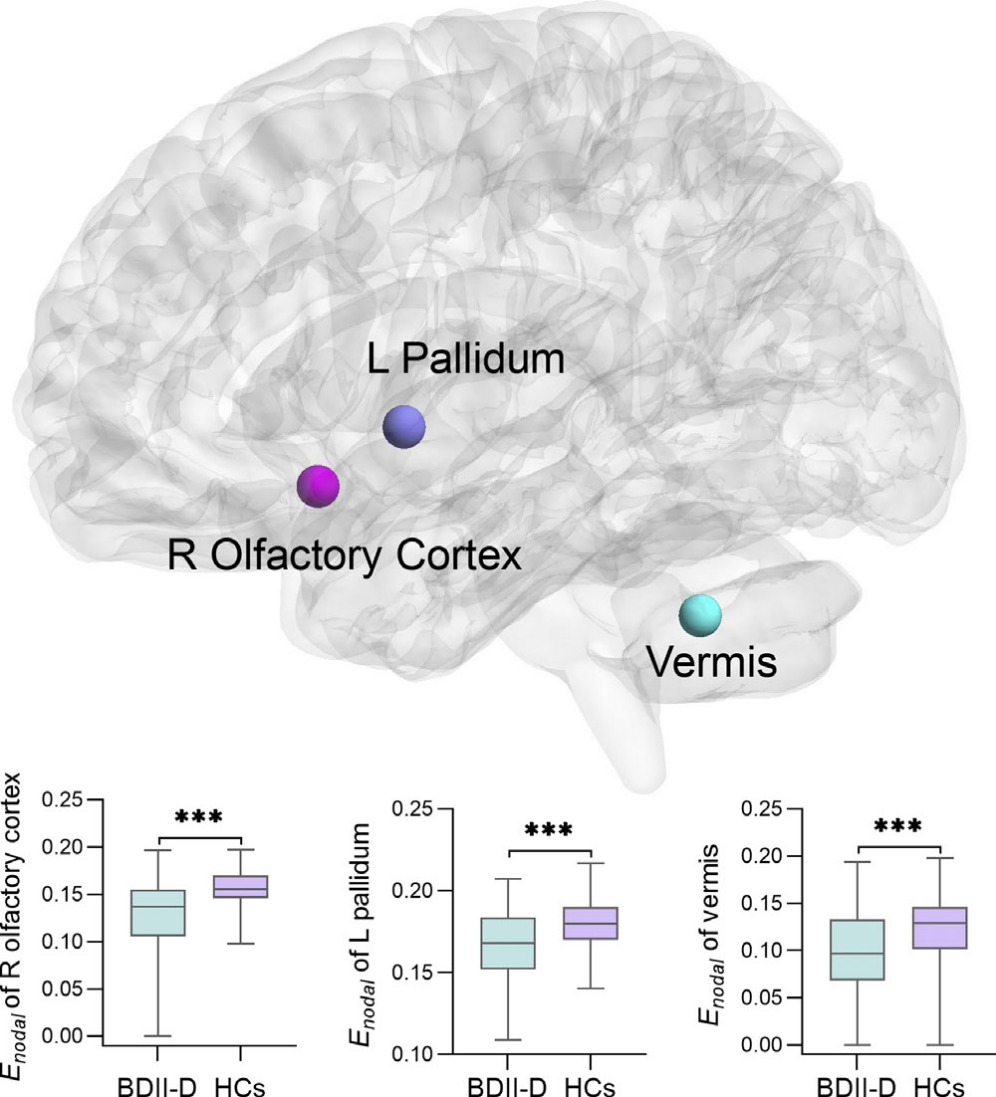
Results of between group differences of the nodal parameter (*E_nodal_*) for the BDII-D and HCs group. The BDII-D patients showed significantly decreased *E_nodal_* in the right olfactory cortex, left pallidum and the vermis (FDR corrected, *p* < 0.05). BDII-D, bipolar II depression; HCs, healthy controls; FDR, false discovery rate; Enodal, nodal efficiency; L, left; R, right. ***: *p* < 0.001.

Figure 2 and Table 2 show the brain regions of significantly different nodal parameters (*E*_nodal_) between the BDII-D group and the HC group. We found decreased *E_nodal_* in the right olfactory cortex (*z* = −3.921, *p* < 0.001, FDR-*p* < 0.001), left pallidum (*t* = −3.628, *p* < 0.001, FDR-*p* < 0.001), and vermis (*z* = −3.523, *p* < 0.001, FDR-*p* < 0.001) in the BDII-D group when compared with the HC group.

### Inflammatory cytokine levels between two groups

The results of comparison of log-transformed cytokine levels are shown in Figure 1 and Table 3. The BDII-D group showed higher levels of IL-8 (*z* = −3.220, *p* = 0.001, FDR-*p* = 0.009), IL-10 (*z* = −3.698, *p* = <0.001, FDR-*p* = <0.001), G-CSF (*z* = −2.664, *p* = 0.008, FDR-p = 0.031), MCP-1 (*t* = −2.689, *p* = 0.009, FDR-*p* = 0.031), and MIP-1β (*t* = −2.773, *p* = 0.008, FDR-*p* = 0.031) compared with the HC group.

**Table 3.** Group differences of 17 cytokine levels between the BDII-D and HC groups

	BD	HCs	*t/z*	*p* values	FDR*-p*
Log IL–1β	−0.10 [−0.14, −0.07]	−0.10 [−0.14, −0.08]	−1.438	0.150^a^	0.232^a^
Log IL–2	0.67 [0.61, 0.73]	0.64 [0.61, 0.66]	−1.987	0.047^a^	0.114^a^
Log IL–4	−0.01 [−0.09, −0.01]	−0.05 [−0.09, −0.01]	−1.476	0.140^a^	0.232^a^
Log IL–5	1.15 [1.09, 1.20]	1.12 [1.07, 1.17]	−0.793	0.428^a^	0.485^a^
Log IL–6	0.41 [0.33,0.45]	0.36 [0.31,0.42]	−1.079	0.281^a^	0.398^a^
Log IL–7	1.02 [0.99,1.05]	1.02 [0.99,1.02]	−0.868	0.386^a^	0.477^a^
Log IL–8	1.04[0.89,1.35]	0.86 [0.81,0.94]	−3.220	0.001^a^	**0.009** ^a^
Log IL–10	0.87 [0.81,0.96]	0.79 [0.76,0.84]	−3.698	<0.001^a^	**<0.001** ^a^
Log IL–12	0.90 [0.84,0.92]	0.88 [0.84,0.92]	−0.855	0.393^a^	0.477^a^
Log IL–13	0.37 [0.32,0.37]	0.34 [0.32,0.37]	−0.177	0.859^a^	0.859^a^
Log IL–17	0.88 [0.87,0.91]	0.88 [0.87,0.91]	−0.259	0.796^a^	0.846^a^
Log G-CSF	1.74 [1.70,1.79]	1.72 [1.69,1.72]	−2.664	0.008^a^	**0.031** ^a^
Log GM-CSF	0.51 [0.47,0.56]	0.47 [0.47,0.54]	−1.550	0.121^a^	0.229^a^
Log IFN-γ	0.53(0.08)	0.48(0.08)	−2.185	0.033^b^	0.094^b^
Log MCP–1	1.47(0.23)	1.34(0.15)	−2.689	0.009^b^	**0.031^b^**
Log MIP–1β	1.66(0.28)	1.48(0.22)	−2.773	0.008^b^	**0.031^b^**
Log TNF-α	1.32 [1.22,1.40]	1.26 [1.22,1.32]	−1.717	0.086^a^	0.183^a^

*Note:* Means (with standard deviations in parentheses) and median [25% quartile, 75% quartile] are reported unless otherwise noted. BDII-D, bipolar II depression; HCs, healthy controls; FDR, false discovery rate; IL-1β, interleukin 1β; IL-2, interleukin 2; IL-4, interleukin 4; IL-5, interleukin 5; IL-6, interleukin 6; IL-7, interleukin 7; IL-8, interleukin 8; IL-10, interleukin 10; IL-12, interleukin 12; IL-13, interleukin 13; IL-17, interleukin 17; G- CSF, granulocyte colony-stimulating factor; GM-CSF, granulocyte-macrophage colony-stimulating factor; IFN-γ, interferon-γ; MCP-1(MCAF), monocyte chemoattractant protein-1 (monocyte chemotactic and activating factor); MIP-1β, macrophage inflammatory protein 1β; and TNF-α, tumor necrosis factor alpha. Bold entries indicate statistical significance at *p* < 0.05 after FDR correction for multiple comparisons.

a The *p* values were obtained by Mann–Whitney *U* tests. Log, base 10 log-transformed.

b The *p* values were obtained by independent sample *t*-tests.

### Correlation analyses

**Figure 3.**
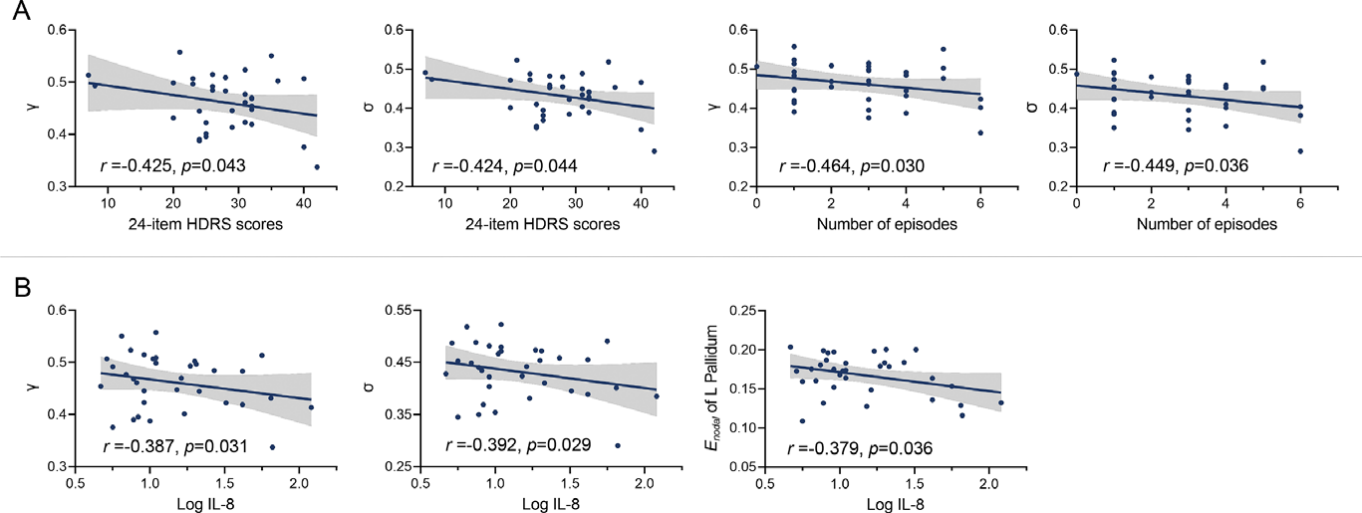
Correlation results between clinical variables, functional network topology and inflammatory cytokine levels. (A) The correlations between abnormal global parameters and 24-item HDRS scores and number of episodes in BDII-D. (B) The correlations between abnormal global and nodal parameters and log IL-8 (base 10 log-transformed) in BDII-D. BDII-D, bipolar II depression; *γ*, normalized clustering coefficient; *σ*, small-worldness; *E_nodal_*, nodal efficiency; L, left; HDRS, Hamilton Depression Rating Scale.

For the correlations between abnormal global parameters and clinical variables, 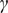 (*r* = −0.425, *p* = 0.043) and 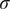 (*r* = −0.424, *p* = 0.044) were negatively correlated with the 24-item HDRS scores. Besides, 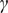 (*r* = −0.464, *p* = 0.030) and 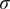 (*r* = −0.449, *p* = 0.036) were negatively correlated with number of episodes in BDII-D (Figure 3A). The non-significant results are shown in Table S1 of the Supplementary Materials.

For the correlations between abnormal inflammatory cytokine levels (log-transformed) and functional network topology in BDII-D, IL-8 levels were negatively correlated with global parameters including 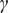 (*r* = −0.387, *p* = 0.031) and 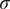 (*r* = −0.392, *p* = 0.029), and negatively correlated with *E_nodal_* of the left pallidum (*r* = −0.379, *p* = 0.036) in BDII-D (Figure 3B). The remaining correlation results in the BDII-D and HC groups are shown in Tables S2–S3 of the Supplementary Materials.

### Validation results

Table S4 of the Supplementary Materials lists the statistical results for the global parameters of weighted network between the BDII-D group and the HC group. The BDII-D group showed significantly decreased 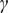 (*t* = −3.266, *p* = 0.001, FDR-*p* = 0.004) and 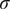 (*t* = −3.756, *p* < 0.001, FDR-*p* < 0.001) compared to HCs. These results indicate that the results of global parameters including normalized clustering coefficient 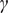 and small-worldness 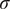 reported above were relatively reliable across the different network type strategies.

Figure S1 of the Supplementary Materials shows the robustness of the correlation results between clinical variables, weighted functional network topological parameters, and inflammatory cytokine levels. The correlations between abnormal global parameters including 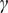 (*r* = −0.426, *p* = 0.034) and 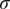 (*r* = −0.483, *p* = 0.017) and the 24-item HDRS scores in BDII-D, the correlations between abnormal global parameters including 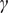 (*r* = −0.399, *p* = 0.048) and 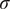 (*r* = −0.423, *p* = 0.040) and number of episodes in BDII-D, and the correlations between abnormal global parameters including 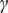 (*r* = −0.377 *p* = 0.037) and 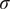 (*r* = −0.409, *p* = 0.022) and IL-8 level in BDII-D still exist in the validation analyses.

## Discussion

The main findings of the present study are as follows: (1) altered global parameters including greater normalized characteristic path length *λ*, lower normalized clustering coefficient 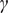 , small-worldnes 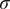 , global efficiency *E*_glob,_ and local efficiency *E*_loc_ in unmedicated BDII-D; (2) decreased nodal efficiency *E*_nodal_ in the right olfactory cortex, left pallidum, and vermis in unmedicated BDII-D; (3) elevated levels of IL-8, IL-10, and G-CSF in unmedicated BDII-D; (4) elevated IL-8 level was negatively correlated with decreased 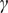 and 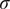 , and *E_nodal_* of the left pallidum in BDII-D; and (5) decreased 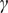 and 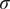 were correlated with higher HDRS scores in BD. This study is among the first to investigate the relationship between functional network topology and inflammation markers in unmedicated BDII-D individuals. Notably, the highly homogeneous samples included in this study are unmedicated individuals with BDII-D, eliminating confounding factors such as medication, BD subtypes, and mania or hypomania state, which may provide more reliable evidence of the neuropathological mechanisms behind depressive symptoms of BD individuals from the perspective of inflammation.

In this study, the whole-brain functional networks in BDII-D and HC groups showed small-world properties that were consistent with previous findings (Bassett & Bullmore, 2006; Suo et al., 2018). Additionally, we found significant decreased 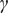 , 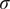 , *E*_glo,_ and *E*_loc_, and significant increased *λ* in BD, which partly validate the topological disruption among BDII-D in our previous study (Wang et al., 2017) with a larger sample size and different templates for constructing brain network, and further suggest its correlation with inflammation. Global efficiency *E*_glo_ quantifies parallel information transfer capacity across distributed networks, while local efficiency *E*_loc_ reflects specialized processing within modular subsystems (Bullmore & Sporns, 2009). Characteristic path length λ inversely correlates with integration efficiency, with higher values indicating suboptimal long-range communication. Small-world coefficients (σ = γ/λ) balance local segregation and global integration – a configuration evolutionarily optimized for cognitive flexibility (Bassett & Sporns, 2017). The observed changes in network parameters collectively suggest that the brain’s functional organization in BD is characterized by impaired segregation and integration (Yoon, Kim, Kim, & Lyoo, 2021), which is supported by some previous graph theoretical studies using rs-fMRI in BD (Spielberg et al., 2016; Wang et al., 2017; Yu et al., 2020). These functional network topological abnormalities might lead to impaired cognitive integration, disrupted local processing, and reduced network flexibility, which in turn contribute to emotional dysregulation, executive dysfunction, and cognitive rigidity in BD. Notably, we found decreased 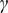 and 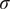 were correlated with the HDRS scores and number of episodes in BDII-D, suggesting more serious depressive symptoms and more episodes are related to more pronounced disrupted functional networks in unmedicated BDII-D. Specifically, a previous rs-fMRI study reported that altered resting-state functional network connectivity was significantly associated with symptom severity on depression rating scales in unmedicated BD (He et al., 2016). However, this previous study had the limitation of a relatively small sample size and inclusion of both BD type I and type II individuals. In the context of cognitive therapy, brain functional network change was associated with the improvement of emotion regulation in mood-dysregulated adolescents at familial risk for BD (Qin et al., 2021), which suggests abnormal brain functional network might underline the mechanism of mood dysregulation. Furthermore, we found the correlation between brain functional network topology and number of episodes in BD. Although few studies have reported this correlation in BD, studies in MDD have reported the number of depressive episodes was significantly positively associated with altered functional network connectivity (Goya-Maldonado et al., 2016; Lu et al., 2023; Morgan et al., 2016). Therefore, our findings shed lights on the importance of functional network topological abnormalities in the neuropsychological processes and neuropsychiatric symptoms of BD.

At the nodal level, the BDII-D group showed lower nodal efficiency in the left pallidum, right olfactory cortex, and cerebellum vermis, suggesting impaired regional connectivity of these regions. These findings were partly consistent with previous studies showing decreased nodal efficiency of the left pallidum (A. Zhang et al., 2023) and lower nodal characteristic path length in the right olfactory cortex in BD (Dvorak et al., 2019). Emerging evidences proved the basal ganglia including the pallidum regulate affect (Caligiuri et al., 2006) and carry non-motor information such as reward and sensory stimuli (Howell et al., 2016). In healthy individuals, reduced incentive cue-related activation in the pallidum was associated with higher anhedonia (Chung & Barch, 2015), which is a core symptom of depression (Pizzagalli, 2014). Also, decreased cerebral blood flow was found in the left pallidum in adolescents with depression (Fu et al., 2023). The olfactory cortex has been reported to be involved in sensory functions, emotional regulation, and memory formation (Yuan & Slotnick, 2014). In animal models, bilateral olfactory bulbectomy results in changes in behavior, and in the endocrine, immune, and neurotransmitter systems that simulate many of those seen in individuals with depression (Coppola & Parrish Waters, 2021; Song & Leonard, 2005). Neuroimaging studies have demonstrated structural abnormalities of the olfactory sulci in BD (Kocakaya, Bayar Muluk, & Bekin Sarikaya, 2023; Takahashi et al., 2014). The vermis is considered to be a part of the limbic cerebellum (Bodranghien et al., 2016; Schmahmann, 2004), which is critical in the modulation of cognition and emotion (Schmahmann, Weilburg, & Sherman, 2007). Several previous studies have reported decreased amplitude of low-frequency fluctuation (Chen et al., 2022; Gong et al., 2020; Lai et al., 2019; Shunkai et al., 2023; Zhao et al., 2020) in the cerebellum in unmedicated BD II. A recent study has demonstrated that the baseline functional connectivity between the vermis and temporal lobes was associated with antidepressant treatment outcomes in individuals with mood disorders (Wang et al., 2023). On the basis of these studies, this study further shows the important roles the pallidum, olfactory cortex, and vermis play in the neurophysiological mechanism of BDII-D.

In this study, we found significantly elevated inflammatory cytokine levels such as IL-8, IL-10, and G-CSF in BDII-D, which were largely consistent with previous findings in meta-analyses of BD (Misiak et al., 2020; Modabbernia et al., 2013). Notably, we found a negative correlation between IL-8 and functional brain network topology in BD, suggesting that inflammation might play a role in the shift of functional network topology toward randomization in BD. Previous neuroimaging studies have shown the relationship between inflammation and regional functional connectivity abnormalities in BD (*Chen et al., 2020*; Tang et al., 2021; Tseng et al., 2021). As a continuation and complement to previous research, this study further reflects the involvement of inflammation in the mechanisms of BD from the perspective of the whole-brain functional network. IL-8, a proinflammatory cytokine produced by many cell types including macrophage and microglia, mainly functions as a neutrophil chemoattractant not only in the bloodstream but also in the brain, and represents chronic inflammatory changes in neurodegenerative and neuropsychological alterations in the brain (Tsai, 2021). According to previous evidence, chronic neuroinflammation caused by the activation of microglia and astrocytes in the brain contributes to neuronal loss and disease progression in neurodegenerative diseases (Basurco et al., 2023). Also, neuroinflammation may disrupt the delicate balance needed for neurophysiological activities and exert direct harmful impacts on neural plasticity and neurogenesis, facilitating many kinds of neuropathologies associated with neuropsychiatric diseases including BD (Yirmiya & Goshen, 2011). In cell culture studies, reactive microglia can inhibit neuronal autophagy (Festa et al., 2023) and induce different levels of neuronal network dysfunction (Schilling et al., 2021); GM-CSF induces the proliferation of microglia and disturbs electrical neuronal network rhythms in situ (Dikmen et al., 2020). In animal studies, inflammation at early developmental stages is sufficient to exert a long-lasting effect on glutamatergic synaptogenesis and brain connectivity (Mirabella et al., 2021), and targeting neuroinflammation by pharmacologic downregulation of inflammatory pathways is neuroprotective in protein misfolding disorders (Risen et al., 2024). In individuals with depression, inflammation was associated with reduced functional connectivity in a widely distributed network centralized in the ventral medial prefrontal cortex (Yin et al., 2019). A recent systematic review has shown the association of blood biomarkers including IL-8 with cerebral white matter and myelin content in BD (Ali, Husnudinov, Wollenhaupt-Aguiar, & Frey, 2024). Interestingly, a recent study shows that regional global brain connectivity changed after pro- and anti-inflammatory therapies (Martins et al., 2022). Based on the previous findings, the potential influence of IL-8 on brain network topology may be mediated through the activation of microglia and the subsequent disruption of neurotransmitter metabolism. These processes could exert both direct and indirect effects on neural network architecture. Specifically, the activation of microglia by IL-8 may lead to the release of pro-inflammatory cytokines, which in turn can impair neuronal function, disrupt neural connectivity, and attenuate neuroplasticity. Collectively, these alterations may contribute to the observed changes in brain network topology, highlighting the multifaceted role of IL-8 in modulating neuroinflammatory processes and their downstream effects on neural network integrity. Future research is needed to further elucidate the mechanisms underlying the role of IL-8 in neuroinflammation and to explore its potential as a biomarker and therapeutic target.

Furthermore, in this study, we found elevated IL-8 level was correlated with decreased nodal efficiency in the left pallidum in BDII-D, which suggests inflammation might contribute to impaired regional connectivity in the basal ganglia in BD. Decreased nodal efficiency in the left pallidum has been previously reported in BD individuals (Zhang et al., 2023). In the present study, we validated this finding in the BDII-D subtype and further revealed a potential association between this neuroimaging alteration and peripheral inflammation. Emerging evidences have demonstrated that inflammatory cytokines are associated with abnormal basal ganglia function and neurotransmitter activities, leading to symptoms of depression. For example, in nonhuman primates, a positron emission tomography study indicated that chronic peripheral cytokine exposure reduces striatal dopamine release in association with anhedonia-like behavior (Felger et al., 2013). In individuals with major depression, increased inflammation is associated with increased basal ganglia glutamate activity (Haroon et al., 2016). Also, the administration of the inflammatory cytokine has been shown to increase brain glutamate in the basal ganglia as measured by magnetic resonance spectroscopy in depression (Haroon & Miller, 2017). Besides, inflammation was consistently found to affect basal ganglia and cortical reward and motor circuits to drive reduced motivation and motor activity, which may result from cytokine effects on monoamines and glutamate (Felger, 2018). A task-fMRI study showed the associations between inflammatory processes and activation of basal ganglia regions during multiple phases of reward processing in psychotropic medication-free youth with psychiatric symptoms (Bradley et al., 2019). Based on the previous findings, our result suggests that the pallidum may serve as a potential neuroimaging biomarker and anti-inflammation target in BD in future research. Moreover, the role of IL-8 in neuroinflammation highlights its potential as a therapeutic target. By modulating IL-8 signaling, it may be possible to reduce neuroinflammatory processes that contribute to neuronal dysfunction and network abnormalities, as observed in conditions like BD. Targeting IL-8 could also help in mitigating the downstream effects of chronic inflammation on brain connectivity and plasticity, potentially improving clinical outcomes.

The association between aberrant network topology and IL-8 level underscores the necessity of investigating IL-8 as a therapeutic target to restore normal brain network function and enhance clinical outcomes in individuals with BDII-D. Additionally, the correlation between abnormal brain network topology and the HDRS scores and number of episodes in BDII-D suggests the potential to develop personalized treatment strategies that address both inflammation and network connectivity, ultimately improving symptom management and reducing the recurrence of depressive episodes in BDII-D individuals.

## Limitations

There are several limitations to this study that need to be mentioned. First, the sample size was relatively small. Additional studies with a bigger sample size from multi-centric cohorts or data-sharing initiatives are needed in future research. Second, since this was a cross-sectional study, we were unable to further investigate the causal relationship between topological organization disruption and inflammation. Therefore, longitudinal studies are necessary to confirm the role of cytokines in brain function. Third, we were unable to collect the cytokine levels in CSF in this study. However, peripheral cytokine levels remain the primary basis for evaluation due to ethical considerations for acquiring CSF or brain tissue. Peripheral cytokine levels may not fully reflect brain cytokine levels, but may affect brain cytokine production. Besides, cytokine levels may also be influenced by stress. More research is needed to explore the role of pathological “states” (i.e. current mental disorders) and their impact on brain immune relationships under socially stressful conditions. Fourth, the correlation results did not withstand after multiple comparison corrections. Therefore, our findings in this study should be regarded as preliminary and should be cautiously inferred, which should be further replicated in a larger sample size or multi-center study. Fifth, chronic inflammation is known to be associated with a wide range of metabolic and immune changes, which could potentially confound the interpretation of our results. Future studies should consider incorporating more comprehensive assessments of inflammatory status to better control for this potential confounding factor.

## Conclusion

To summarize, this study displayed disrupted brain functional network topology and elevated peripheral cytokines in BD. Elevated pro-inflammatory cytokine IL-8 level might contribute to both decreased global brain network integration and segregation, and regional efficiency of the basal ganglia in unmedicated BDII-D.

## Supporting information

10.1017/S0033291725100834.sm001 Tang et al. supplementary material Tang et al. supplementary material

## Data Availability

The data that support the findings of this study are available from the corresponding author, upon reasonable request.

## Supplementary material

The supplementary material for this article can be found at http://doi.org/10.1017/S0033291725100834.
