# Supplementary material for: Disrupted brain functional network topology is associated with peripheral inflammation in unmedicated bipolar II depression
Source: Psychol Med. 2026 Apr 1;56:e86. doi: 10.1017/S0033291725100834 (PMC13079219; doi:10.1017/S0033291725100834)
Supplement: Tang et al. supplementary material [file S0033291725100834sup001.docx]

***Supplementary Materials***

**Disrupted brain functional network topology is associated with peripheral inflammation in unmedicated bipolar II depression**

**Inflammatory cytokines measures**

Blood samples from BDII-D and HCs were collected in the morning under fasting conditions, with participants abstaining from alcoholic beverages for at least one day prior to testing. Four milliliters of fasting blood were drawn from each subject via venipuncture into sterile Vacutainer tubes (Becton & Dickinson 367812; Becton Dickinson, Franklin Lakes, NJ, USA). The samples were then left to clot for a period of 30 minutes before being centrifuged at 1,000 g for 15 minutes at a temperature of 4 °C. Then, the serum was subjected to a second round of centrifugation at 3,000 rpm for 10 minutes, also at 4 °C, and stored at -80 °C until analysis. The serum was strictly required to melt at low temperature on the ice before detection, and the detection processes were carried out at room temperature as required by the kit to ensure the effective binding of antigen and antibody. The average intra-assay coefficients of variation (CVs) were <10% for all analyzed inflammatory cytokines. No cytokines were below the limits of assay detection.

**Table S1. Correlation results between abnormal network parameters, abnormal inflammatory cytokines levels and clinical variables in BDII-D group.**

|  |  | **Inflammatory cytokines** | | | | |  | **Global parameters** | | | | |  | **Nodal efficiency** | | |
| --- | --- | --- | --- | --- | --- | --- | --- | --- | --- | --- | --- | --- | --- | --- | --- | --- |
|  |  | Log IL-8 | Log  IL-10 | Log  G-CSF | Log MCP-1 | Log MIP-1β |  | *γ* | *λ* | *σ* | *Eglo* | *Eloc* |  | R Olfactory Cortex | L Pallidum | Vermis |
| Age at onset (years) | r | 0.215 | 0.086 | 0.266 | 0.308 | 0.252 |  | 0.324 | -0.116 | 0.316 | 0.212 | 0.119 |  | 0.321 | 0.106 | -0.011 |
|  | p | 0.245 | 0.647 | 0.149 | 0.092 | 0.172 |  | 0.075 | 0.533 | 0.084 | 0.253 | 0.523 |  | 0.078 | 0.572 | 0.952 |
|  | ν | 29 | 29 | 29 | 29 | 29 |  | 29 | 29 | 29 | 29 | 29 |  | 29 | 29 | 29 |
| Number of episodes | r | 0.138 | 0.007 | -0.098 | -0.130 | 0.042 |  | **-0.464** | 0.233 | **-0.449** | -0.257 | -0.262 |  | **-0.383** | -0.195 | 0.064 |
|  | p | 0.460 | 0.972 | 0.602 | 0.485 | 0.821 |  | **0.030^*^** | 0.207 | **0.036^*^** | 0.162 | 0.155 |  | **0.033^*^** | 0.294 | 0.733 |
|  | ν | 29 | 29 | 29 | 29 | 29 |  | 29 | 29 | 29 | 29 | 29 |  | 29 | 29 | 29 |
| 24-item HDRS score | r | 0.091 | -0.208 | -0.370 | -0.276 | 0.254 |  | **-0.425** | 0.189 | **-0.424** | -0.107 | -0.234 |  | -0.097 | -0.158 | -0.217 |
|  | p | 0.627 | 0.261 | 0.040 | 0.133 | 0.168 |  | **0.043^*^** | 0.308 | **0.044^*^** | 0.566 | 0.205 |  | 0.604 | 0.395 | 0.242 |
|  | ν | 29 | 29 | 29 | 29 | 29 |  | 29 | 29 | 29 | 29 | 29 |  | 29 | 29 | 29 |
| YMRS score | r | 0.122 | -0.071 | -0.116 | 0.177 | -0.152 |  | 0.005 | 0.117 | -0.014 | 0.067 | -0.139 |  | 0.337 | -0.233 | -0.209 |
|  | p | 0.512 | 0.704 | 0.536 | 0.341 | 0.413 |  | 0.980 | 0.532 | 0.942 | 0.721 | 0.457 |  | 0.063 | 0.207 | 0.260 |
|  | ν | 29 | 29 | 29 | 29 | 29 |  | 29 | 29 | 29 | 29 | 29 |  | 29 | 29 | 29 |
| Duration of illness (months) | r | 0.181 | 0.000 | 0.022 | 0.118 | 0.131 |  | 0.003 | 0.055 | -0.007 | 0.027 | -0.104 |  | 0.164 | -0.005 | -0.201 |
|  | p | 0.329 | 1.000 | 0.906 | 0.526 | 0.482 |  | 0.988 | 0.769 | 0.969 | 0.884 | 0.576 |  | 0.379 | 0.980 | 0.277 |
|  | ν | 29 | 29 | 29 | 29 | 29 |  | 29 | 29 | 29 | 29 | 29 |  | 29 | 29 | 29 |

Partial correlation analyses (controlled for age, gender and education) between network properties, inflammatory cytokines levels, and clinical variables in BDII-D. BDII-D, bipolar II depression; HDRS, Hamilton Depression Rating Scale; YMRS, Young Mania Rating Scale; IL-8, interleukin 8; IL-10, interleukin 10; G- CSF, granulocyte colony-stimulating factor; MCP-1, monocyte chemoattractant protein-1; MIP-1β, macrophage inflammatory protein 1β; $\lambda$, normalized characteristic path length; $\gamma$, normalized clustering coefficient; *σ, small world*; *E_glo_*, global efficiency; *E_loc_*, local efficiency; R, right; L, left; **^*^**, *p* < 0.05. *ν,* degrees of freedom. The number of independent tests for the partial correlation analysis is 65. Log, base 10 log-transformed.

**Table S2. Correlation results between abnormal network parameters and inflammatory cytokines levels in BDII-D group.**

|  |  | **Global parameters** | | | | |  | **Nodal efficiency** | | |
| --- | --- | --- | --- | --- | --- | --- | --- | --- | --- | --- |
|  |  | *γ* | *λ* | *σ* | *E_glo_* | *El_oc_* |  | R Olfactory Cortex | L Pallidum | Vermis |
| Log IL-8 | *r* | **-0.387** | 0.340 | **-0.392** | -0.279 | -0.112 |  | -0.098 | **-0.379** | -0.312 |
|  | *p* | **0.031^*^** | 0.062 | **0.029^*^** | 0.129 | 0.547 |  | 0.600 | **0.036^*^** | 0.087 |
|  | *ν* | 29 | 29 | 29 | 29 | 29 |  | 29 | 29 | 29 |
| Log IL-10 | *r* | -0.348 | 0.193 | -0.331 | **-0.388** | -0.279 |  | **-0.391** | -0.230 | -0.198 |
|  | *p* | 0.055 | 0.297 | 0.069 | **0.031**^*^ | 0.129 |  | **0.030^*^** | 0.213 | 0.285 |
|  | *ν* | 29 | 29 | 29 | 29 | 29 |  | 29 | 29 | 29 |
| Log G-CSF | *r* | -0.150 | 0.157 | -0.157 | -0.175 | -0.056 |  | -0.307 | 0.135 | 0.047 |
|  | *p* | 0.420 | 0.399 | 0.399 | 0.346 | 0.763 |  | 0.093 | 0.469 | 0.800 |
|  | *ν* | 29 | 29 | 29 | 29 | 29 |  | 29 | 29 | 29 |
| Log MCP-1 | *r* | -0.121 | 0.169 | -0.125 | -0.159 | 0.002 |  | -0.018 | -0.062 | -0.023 |
|  | *p* | 0.517 | 0.365 | 0.502 | 0.392 | 0.992 |  | 0.923 | 0.739 | 0.901 |
|  | *ν* | 29 | 29 | 29 | 29 | 29 |  | 29 | 29 | 29 |
| Log MIP-1β | *r* | -0.311 | 0.279 | -0.307 | -0.323 | -0.191 |  | -0.187 | -0.330 | **-0.373** |
|  | *p* | 0.089 | 0.128 | 0.093 | 0.076 | 0.302 |  | 0.313 | 0.070 | **0.039^*^** |
|  | *ν* | 29 | 29 | 29 | 29 | 29 |  | 29 | 29 | 29 |

Partial correlation analyses (controlled for age, gender and education) between network properties, and inflammatory cytokines levels in BDII-D group. BDII-D, bipolar II depression; IL-8, interleukin 8; IL-10, interleukin 10; G- CSF, granulocyte colony-stimulating factor; MCP-1, monocyte chemoattractant protein-1; MIP-1β, macrophage inflammatory protein 1β; $\lambda$, normalized characteristic path length; $\gamma$, normalized clustering coefficient; *σ, small world*; *E_glo_*, global efficiency; *E_loc_*, local efficiency; R, right; L, left; **^*^**, *p* < 0.05.  *ν,* degrees of freedom. The number of independent tests for the partial correlation analysis is 40. Log, base 10 log-transformed.

**Table S3. Correlation results between network parameters and inflammatory cytokines levels in HCs group.**

|  |  | **Global parameters** | | | | |  | **Nodal efficiency** | | |
| --- | --- | --- | --- | --- | --- | --- | --- | --- | --- | --- |
|  |  | *γ* | *λ* | *σ* | *E_glo_* | *El_oc_* |  | R Olfactory Cortex | L Pallidum | Vermis |
| Log IL-8 | *r* | 0.041 | 0.161 | 0.010 | 0.334 | -0.059 |  | **-0.383** | -0.125 | **0.426** |
|  | *p* | 0.841 | 0.423 | 0.961 | 0.089 | 0.770 |  | **0.049^*^** | 0.534 | **0.027^*^** |
|  | *ν* | 25 | 25 | 25 | 25 | 25 |  | 25 | 25 | 25 |
| Log IL-10 | *r* | 0.299 | -0.325 | 0.345 | 0.257 | 0.189 |  | -0.331 | 0.133 | **0.447** |
|  | *p* | 0.129 | 0.098 | 0.078 | 0.196 | 0.344 |  | 0.092 | 0.510 | **0.020^*^** |
|  | *ν* | 25 | 25 | 25 | 25 | 25 |  | 25 | 25 | 25 |
| Log G-CSF | *r* | -0.105 | -0.016 | -0.101 | 0.028 | 0.126 |  | -0.039 | 0.076 | 0.086 |
|  | *p* | 0.603 | 0.938 | 0.615 | 0.888 | 0.531 |  | 0.848 | 0.707 | 0.670 |
|  | *ν* | 25 | 25 | 25 | 25 | 25 |  | 25 | 25 | 25 |
| Log MCP-1 | *r* | -0.097 | -0.014 | -0.097 | -0.248 | 0.014 |  | -0.214 | 0.144 | 0.214 |
|  | *p* | 0.632 | 0.945 | 0.631 | 0.213 | 0.944 |  | 0.284 | 0.475 | 0.283 |
|  | *ν* | 25 | 25 | 25 | 25 | 25 |  | 25 | 25 | 25 |
| Log MIP-1β | *r* | -0.292 | **-0.383** | -0.225 | -0.075 | -0.176 |  | -0.313 | 0.177 | 0.056 |
|  | *p* | 0.139 | **0.048^*^** | 0.260 | 0.710 | 0.379 |  | 0.112 | 0.376 | 0.782 |
|  | *ν* | 25 | 25 | 25 | 25 | 25 |  | 25 | 25 | 25 |

Partial correlation analyses (controlled for age, gender and education) between network properties, and inflammatory cytokines levels in HCs. HCs, healthy controls; IL-8, interleukin 8; IL-10, interleukin 10; G- CSF, granulocyte colony-stimulating factor; MCP-1, monocyte chemoattractant protein-1; MIP-1β, macrophage inflammatory protein 1β; $\lambda$, normalized characteristic path length; $\gamma$, normalized clustering coefficient; *σ, small world*; *E_glo_*, global efficiency; *E_loc_*, local efficiency; R, right; L, left; **^*^**, *p* < 0.05. *ν,* degrees of freedom. The number of independent tests for the partial correlation analysis is 40. Log, base 10 log-transformed.

**Table S4.** **Statistical results for the global parameters of weighted network between BDII-D group and HCs group**

| Parameters | BD | HCs | ***t//z*** | ***p* values** | **FDR-***p* |
| --- | --- | --- | --- | --- | --- |
| *γ* | 0.4779 (0.0589) | 0.5096 (0.0450) | -3.266 | 0.001^*^ | **0.004^*^** |
| *λ* | 0.3380 [0.3343-0.3434] | 0.3355 [0.3307-0.3412] | -1.811 | 0.070^#^ | 0.113^#^ |
| *σ* | 0.4204 (0.0525) | 0.4520 (0.0376) | -3.756 | <0.001^*^ | **<0.001^*^** |
| *E_glo_* | 0.0959 [0.0892-0.1033] | 0.0940 [0.0890-0.1013] | -0.790 | 0.430^#^ | 0.502^#^ |
| *E_loc_* | 0.1357 [0.1263-0.1460] | 0.1319 [0.1219-0.1397] | -1.743 | 0.081^#^ | 0.113^#^ |

Means (with standard deviations in parentheses) and median [25% quartile-75% quartile] are reported unless otherwise noted. BDII-D, bipolar II depression; HCs, healthy controls; FDR, false discovery rate; $\lambda$, normalized characteristic path length; $\gamma$, normalized clustering coefficient; *σ, small world*; *E_glo_*, global efficiency; *E_loc_*, local efficiency. *The *p* values were obtained by independent-sample *t*-tests. #The *p* values were obtained by Mann-Whitney *U* test.


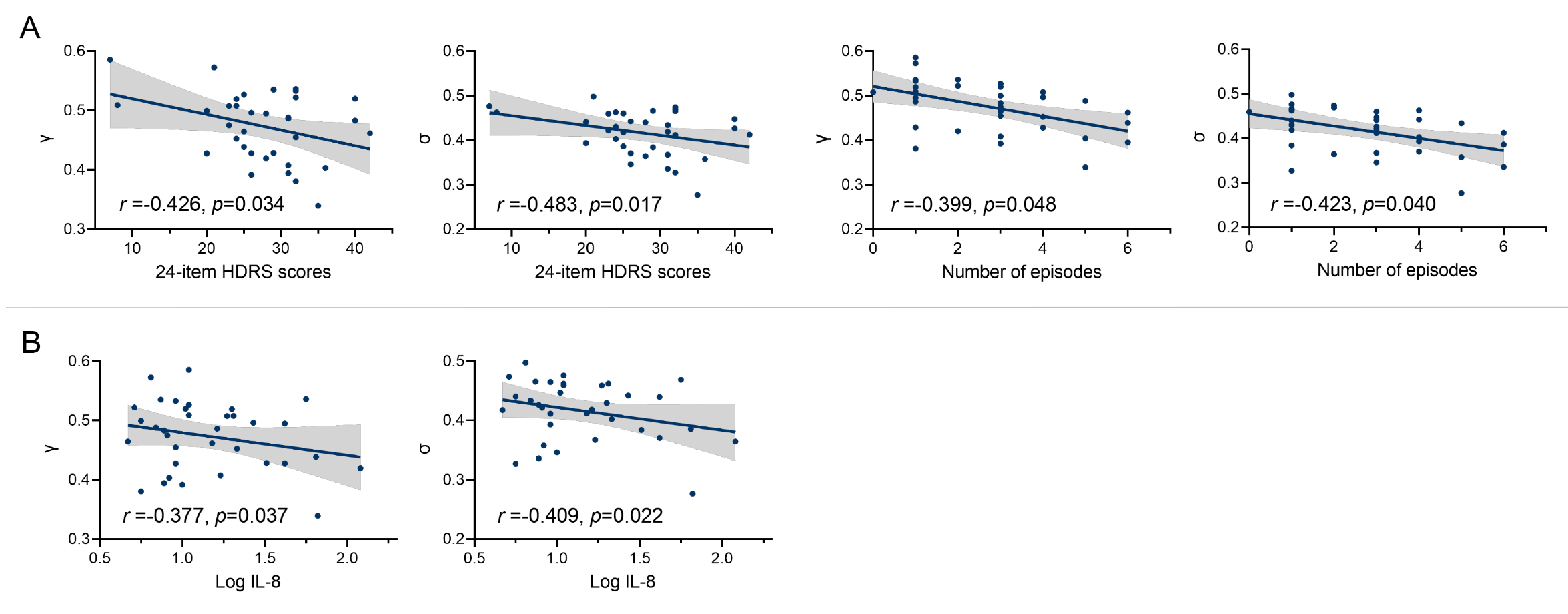


**Figure S1.** **Correlation results between clinical variables, weighted functional network topological parameters and inflammatory cytokine levels.** (A) The correlations between abnormal global parameters and 24-item HDRS, and number of episodes in BDII-D. (B) The correlations between abnormal global parameters and log IL-8 (base 10 log-transformed) in BDII-D.
